# Shift in distribution of division of labour in chronically stressed honeybee colonies after perturbation

**DOI:** 10.1242/jeb.247976

**Published:** 2024-11-08

**Authors:** Zeynep N. Ulgezen, Coby van Dooremalen, Frank van Langevelde

**Affiliations:** ^1^ Wageningen Plant Research, Wageningen University & Research, Droevendaalsesteeg 1, 6708 PB Wageningen, The Netherlands; ^2^ Wildlife Ecology and Conservation Group, Department of Environmental Sciences, Wageningen University & Research, Droevendaalsesteeg 3a, 6708 PB Wageningen, The Netherlands

**Keywords:** *Apis mellifera*, Foragers, Middle-aged bees, Nurses, Task allocation, *Varroa destructor*

## Abstract

Division of labour (DOL) in eusocial insects plays an important role in colony fitness. Honeybees face a variety of stressors that compromise the homeostasis of the colony and reduce survival and reproduction. Considering the significance of DOL in colony homeostasis, it is important to understand whether and how DOL may be altered as a result of chronic stress. Therefore, we tested whether honeybee colonies shift DOL in response to high infestation with the parasitic mite *Varroa destructor*. For this, we monitored chronically stressed and presumably low-stress colonies from April till December 2022. During the experiment, we applied a cold shock to test whether a perturbation resulted in a larger alteration in DOL in chronically stressed colonies. We found that after cold shock, there was a lower proportion of nurses in the chronically stressed colonies. For foragers, we found higher activity post-cold shock in chronically stressed colonies, but no difference between treatments in nectar inflow, suggesting less efficient foragers. Furthermore, we found that there was an accelerated task switch in chronically stressed colonies after the cold shock. The large changes after the perturbation may indicate inefficient task allocation due to chronic stress. Our study contributes to the understanding of social resilience and chronic stress responses in eusocial animals.

## Teaser

**Summary:** Chronic stress alters task allocation in honeybee colonies, by accelerating worker maturation and reducing worker efficiency and survival, leaving colonies more vulnerable to environmental disturbances.

## INTRODUCTION

Division of labour (DOL) is an important characteristic of eusocial insects, i.e. ants, bees, wasps and termites, which allows task parallelization and specialization to meet colony demands and enhance productivity (Duarte et al., 2012). As with many species, eusocial organisms are exposed to a multitude of stressors. However, their stress response is more complex because of their highly social structure, where beyond the individual, the colony homeostasis may be compromised. A crucial role of DOL is to enable colonies to respond to changes in the internal colony status and the environment (Robinson, 1992). The efficiency of DOL to respond to these changes depends on the (re)allocation of individuals in the colony across the required tasks. Therefore, stressors that reduce the flexibility of DOL may have implications for colony homeostasis (Ulgezen et al., 2021). In honeybees (*Apis mellifera*), while there have been extensive behavioural studies on DOL (see Johnson, 2010), there is a lack of studies investigating the potential consequences of chronic stress on DOL at the colony level. Given the importance of honeybees in pollination for agriculture and biodiversity (Hung et al., 2018; Klein et al., 2007) and considering the alarmingly high losses due to stressors over the past several decades (Bruckner et al., 2023; Genersch et al., 2010), there is a need to improve our knowledge on their stress response.

In honeybees, DOL is characterized by temporal polyethism, where transitioning between tasks is age dependent. Typically, honeybee workers successively switch from cell cleaners (cleaning brood cells), to nurses (mainly tending the brood), middle-aged bees (MABs, which perform various in-hive tasks, such as storing food, nest defence and nest building) and foragers (gathering resources) (Seeley, 1985). Colonies can show a dynamic response to changing conditions, where workers may flexibly shift between tasks, by delaying, accelerating or reversing physiological and/or behavioural responses (Huang and Robinson, 1996). For instance, a change in environmental triggers, such as an increase in pollen availability, may accelerate the rate of task switching from MABs to foragers, by reducing contact between food-receiving MABs and foragers, thereby decreasing the transfer of socially inhibiting forager pheromones (Leoncini et al., 2004), pulling more MABs to the foraging task (Johnson, 2010). Foragers in the colony are also influenced by colony size and the amount of brood (Eckert et al., 1994), where increased levels of brood pheromone can lead to higher foraging activity and younger age of forager recruitment (Le Conte et al., 2001). Likewise, increased levels of brood pheromones accelerate nurse development and behaviour (Mohammedi et al., 1996; Sagili and Pankiw, 2009). When brood size reduces, induced by low pollen availability, worker bees transition to long-living winter bees. As the proportion of workers involved in a task and the rate of task switching may change based on changes in the internal colony status (Huang and Robinson, 1996) and the environment (Fewell and Winston, 1992), honeybees are thought to utilize DOL as a coping mechanism for perturbations (Robinson, 1992; Ulgezen et al., 2021).

Alterations in the allocation of workers over tasks and worker efficiency due to chronic stress could potentially disrupt the effectiveness of DOL as a coping mechanism for perturbations, and diminish social resilience (Ulgezen et al., 2021). Social resilience is the collective capacity of superorganisms to maintain homeostasis and buffer against detrimental effects of perturbations (van Dooremalen et al., 2018). The term was first used to describe the efficient redistribution of workers in ant colonies (*Leptothorax unifasciatus*) after being exposed to a perturbation (Sendova-Franks and Franks, 1994). In honeybees, chronic stressors such as parasites and pathogens can lead to earlier recruitment to tasks (Dussaubat et al., 2013; Janmaat and Winston, 2000; Winston and Fergusson, 1985), possibly as a result of the higher mortality rate in stressed bees (Van Dooremalen et al., 2012), which may reduce social inhibition and lead to accelerated worker development. Several modelling studies show that when colonies are exposed to stressors, the shift towards precocious foragers, which are less efficient and have a higher mortality rate, leads to a rapid loss of workers and colony collapse (Barron, 2015; Perry et al., 2015), suggesting a loss of social resilience. Sudden and severe perturbations can also influence DOL as, for example, simulated heat waves have been found to increase the foraging activity in colonies, presumably due to an increase in the number of foragers (Bordier et al., 2017). Colonies possess a remarkable ability to adapt to changing conditions via DOL (Huang and Robinson, 1996), and can recover from high stress and emergency situations by altering task allocation (van Langevelde et al., 2020). However, chronic stressors may be costly, diminishing the buffering capacity, and leaving colonies more vulnerable to perturbations (Ulgezen et al., 2021).

In this study, we compared DOL in chronically stressed (high *Varroa destructor* infested) colonies with presumably low-stress (control) colonies. To test the possible influence of perturbations on DOL, we exposed all colonies to cold shock. If brood is present when temperature decreases, honeybees are triggered to produce heat to maintain the brood temperature constant, between 33 and 36°C (Fahrenholz et al., 1989). For the production of heat, sufficient bees should be recruited for an energy-demanding task (Stabentheiner et al., 2010). To test whether there was a shift in DOL, we measured the proportion of bees involved in tasks, the rate of task switching, foraging activity and nurse effectivity. *Varroa destructor* is a mite that feeds on fat bodies of honeybees (Ramsey et al., 2019) and is considered to be the most harmful biotic stressor to colonies (Rosenkranz et al., 2010). Mite infestation can impair foragers, by reducing flight ability (Blanken et al., 2015) and return success to the colony (Kralj et al., 2007). Furthermore, it has been found to alter the behaviour of nurse bees (Annoscia et al., 2015), where there is less tending to larvae by nurses and accelerated maturation to foragers (Zanni et al., 2018). Therefore, we expected an earlier task switch and lower proportion of nurses, and thereby a higher proportion of foragers, in chronically stressed colonies compared with control colonies. We also expected that the difference between treatments would be more pronounced after the perturbation, as a result of a loss of resilience in chronically stressed colonies.

## MATERIALS AND METHODS

### Experiment set-up

The fieldwork took place between May and December 2022 at an apiary of Wageningen University & Research (51°57′17.7″N 5°38′12.0″E), The Netherlands. The honeybee colonies (Dutch hybrid of *Apis mellifera* spp.) were supplied by a professional beekeeping company (Inbuzz v.o.f.). For our main experiment, 10 colonies were kept in two-storey wooden (interior dimensions simplex) classical hives (CH) from May till September, and then reduced to one box for the winter season. When there were two boxes, a queen excluder was placed in between, and the queen was kept in the top box. In addition to CH colonies, we also used nine observation hives (OH) with three frames stacked on top of each other. These OH colonies were added to gain higher resolution information on the age of task switching. Observation hives have glass doors on each side which allow for more frequent measurements and the ability to observe worker behaviours with minimal disturbance to colonies. OH colonies were kept from July till October 2022.

To minimize drift between colonies, all colonies (CH and OH) were placed at least 1 m apart, and hives were colour-coded at the flight entrance and grouped according to treatment, with hedges in between as a natural barrier while standardizing the microclimate between sections. At the start of the experiments, colonies had a healthy and young (0–1 years) egg-laying queen, and a standard number of bees. Colonies were restricted from swarming throughout the experiments to prevent the disruption of measurements. Sugar dough was fed to all CH colonies and liquid sugar syrup to all OH colonies *ad libitum*.

### Treatments

Before the start of the experiments, colonies were randomly assigned to either a high *V. destructor* infested group (*Varroa*) or a control group. Each group of the main experiment (CH) consisted of five colonies (*n*=10). For the OH colonies (*n*=9), five colonies were assigned to the *Varroa* group and four colonies to the control group. Control colonies were treated with oxalic acid to keep *V. destructor* infestation low. In January 2022, prior to start of experiments, CH and OH control colonies were treated by trickling oxalic acid when there was no brood (37 g oxalic acid dihydrate in 1 l sugar water, 1:1 w:w ratio for sucrose:water). In the beginning of August 2022, CH control colonies were sprayed with oxalic acid (30 g oxalic acid dihydrate in 1 l water). OH control colonies were sprayed with oxalic acid in June 2022 (30 g oxalic acid dihydrate in 1 l water). Colonies assigned to the *Varroa* group were not treated against mite infestation from the winter prior to the start of experiments until the end. Three CH colonies from the *Varroa* group died in November 2022.

Treatments were validated by quantifying the number of mites in colonies. In all CH colonies, *V. destructor* levels were estimated once a month by counting the naturally falling dead mites on the bottom board of the hives (Dietemann et al., 2013). A week prior to each count, clean bottom boards were placed underneath the colonies. The number of mites was divided by the number of days that the board was left underneath the colony to calculate the daily mite fall. In OH colonies, *V. destructor* levels were estimated by sampling around 50 bees from each colony once a month (August till October). Samples were weighed, shaken in soapy water (dishwasher liquid), rinsed over a sieve with a mesh and then the mites left on the sieve were counted to check for infestation levels (Dietemann et al., 2013). Mite levels of each OH colony were measured as the number of mites per gram bee.

To test the resilience of stressed and control colonies, we applied cold shock as a perturbation in mid-August. The perturbation was for 4 consecutive days, the first 2 days for the CH colonies and the last 2 days for the OH colonies. Each day, 4–5 different hives (randomly assigned from different groups) were placed in a −20°C freezer. CH colonies were placed in the freezer for 16 h between 16:00 h and 08:00 h (the next day). Because of their markedly smaller size, and the more limited insulation provided by the hives, the OH colonies were placed in the freezer for 2 h, between 10:00 h and 12:00 h. Two CH control colonies died as a result of experimental handling pre-cold shock (they overheated while preparing for transport), and measurements post-cold shock were continued with only three CH control colonies.

### Division of labour

Every month, starting from the end of May till October, cohorts of around 100–200 newly emerged bees were marked using a unique colour (Posca marked) and returned to their original CH colony (Van Dooremalen et al., 2012). At 2 week intervals, weather allowing, the number of marked bees from previous cohorts was recorded in each colony. To minimize the disturbance of bees, smoke was not used during measurements. As a further precaution against disturbance, after counts, frames were temporarily moved to a separate brood box (keeping the frame sequence in place), so that the handling of the bees on the frame did not influence the bees remaining in the colony. This was also done to ensure that the possible movement of bees between frames did not affect the results. Each frame side was observed for 1 min, unless there were not many bees on the frame and the marked bees could be easily detected, shortening the time spent per frame side. If a colony was visibly disturbed during the moment of inspection, with a lot of bees flying out, the data were removed from the analysis.

As the location of bees can give information on the type of worker (Seeley, 1982; Wild et al., 2021), bees were either assigned as nurses if they were on the brood nest or as MABs if they were on cells with pollen or nectar. Bees on the entrance of the hive were also counted and recorded as MABs. When bees were on empty frames, their task was recorded as unknown. To look at the proportion of nurses, each measurement week we counted the number of nurses and MABs, and divided the number of nurses by the sum of the number of nurses and MABs. The unique colour assigned to the cohorts was used to determine how many bees had switched tasks and at what age. We also used these data to estimate survival time of individuals from different treatments.

In the OH colonies, cohorts of about 50 newly emerged bees were marked and measured every week. Each week the ages and the tasks of cohorts were recorded. Compared with CH colonies, a more detailed categorization of tasks was done over three task groups: nurses, MABs and foragers. Bees were assigned to one of these task groups based on their behaviours and location in the hive (Table 1).

**Table 1.**
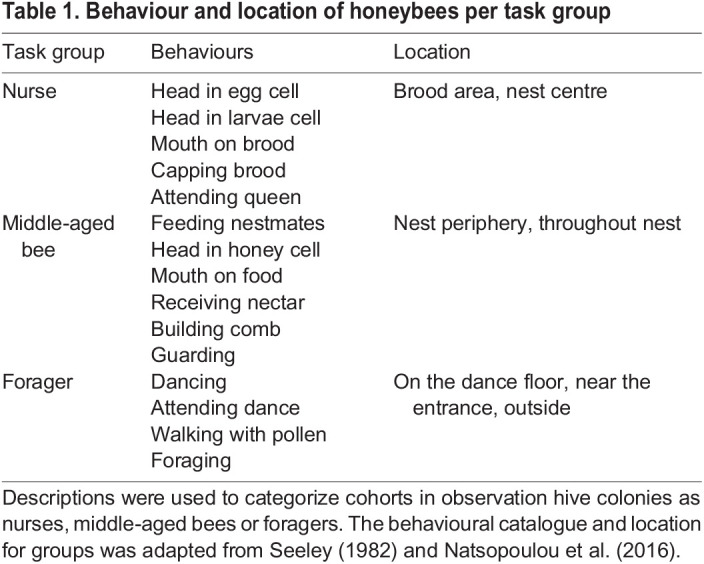
Behaviour and location of honeybees per task group

### Foraging activity and efficiency

For foraging activity and efficiency, we used the mass change of the colonies within a day. All CH colonies were placed on top of a BEEPbase sensor system (https://beep.nl/index.php/measurement-system-2), which included a mass sensor (Bosche H40A, 0–150 kg ±10 g), that logged data every 10 min. Data were transmitted from a long-range (LoRa) gateway and stored in the BEEPapp (https://beep.nl/index.php/beep-app). Using mass data, we looked at foraging activity 7 days prior to and 7 days after the cold shock perturbation (excluding days that the colonies were opened or placed in the freezer). For each day, data were detrended by subtracting the raw mass at each time point from the mass at midnight (Meikle et al., 2018). Only data from dawn, when bees depart the colony, till dusk, when bees return to the colony, were included to capture the active period of foragers. As a proxy for foraging efficiency, we used the daily nectar flow for the same time period as foraging activity. Nectar flow was estimated by using the daily mass change of the colony. Each mass within a day was subtracted from the one preceding it and then summed to calculate the cumulative mass change per day.

All colonies were also equipped with bee counters (bBars, bRemote) at the flight entrance of the hive. Flight activity, i.e. the total number of incoming and outgoing bees, was recorded every 10 min for 30 s. Because of network connectivity issues, there was a high loss of data, especially concerning the period of the cold shock. Therefore, we could not use these data for the analysis of foraging activity. However, we did compare the foraging activity from bee counters with foraging activity derived from mass to confirm that mass data represent foraging activity. To estimate foraging activity from bee counters, we used the mean number of outgoing foragers per day.

### Number of nurses to number of brood cells

To see whether the number of nurses was related to the number of brood cells and whether there was a difference in nurse effectivity between treatments, we measured brood size in CH colonies in July and October. Nurse effectivity was assessed by the nurse load (number of brood cells tended per nurse). The number of brood cells was estimated by placing a grid (5 cm×5 cm squares) over each side of every frame and counting the number of squares that covered the area of brood in all stages (pupae, larvae and eggs). For each colony, the values were summed and a factor of 4 cells per cm^2^ was used to calculate the total number of brood cells (Delaplane et al., 2013).

### Statistical analysis

For treatment validation, in both CH and OH colonies, the difference in mite infestation levels between *Varroa* and control colonies was analysed with a linear mixed model (LMM) including the interaction between treatment and month with colony as subject to account for repeated measurements.

To test whether there was a shift in DOL in stressed colonies, we tested the differences in the proportion of nurses by using LMMs. Treatment, week of the year (to represent time) and their interaction were included as fixed factors and colony as subject for repeated measurements. To investigate the effects of the perturbation on future workforce (in brood at time of cold shock), we did a separate analysis, with proportion of nurses as the dependent variable, and mark week (to represent cohort), weeks alive (to represent age), treatment and their three-way interaction as fixed factors. Assumptions for using linear models were met. Tukey's HSD was used for comparisons between the means of different treatments.

We used a Cox proportional hazards model (CPHM) to calculate the rate of task switching and survival for each cohort of marked bees, and to compare the two treatments. For this, we ran several models with the events: switch to MAB (OH and CH), switch to forager (OH), death (CH). In the analyses for task switching, we removed censored data as they did not necessarily represent the rate of switching, as we did not know the mortality rate for specific task groups. We also did a separate analysis for each dependent variable pre- and post-cold shock to investigate the effect of the perturbation. For all analyses, treatment was used as a fixed factor and the day/week the cohorts were marked (time of emerging from brood) as covariate. For some events, the time of emerging was observed to be time dependent. In such cases, we used a step function in the CPHM to use different coefficients over different time intervals (Therneau et al., 2017).

**Fig. 1.**
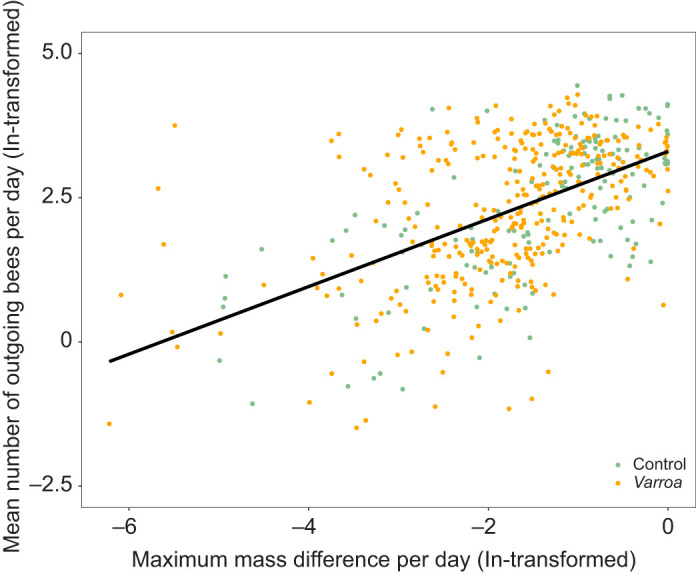
**Correlation between foraging activity from mass and foraging activity from bee counters for the presumably non-stressed (control) and high *Varroa destructor*-infested (*Varroa*) colonies.** Each colony was equipped with a mass sensor and bee counter, and every day data on mass and foraging activity were recorded in 10 min intervals throughout the experimental period in 2022. Foraging activity from mass was estimated as the maximum mass difference from mass at midnight between dusk and dawn. Foraging activity from bee counters was estimated as the mean number of outgoing bees per day. Zero values were removed from the analysis and, because of network connectivity issues, there were gaps in measurements across several days. Both variables were ln-transformed for the analysis (Pearson correlation: *r*=0.53, *n*=492, *P*<0.001).

To compare foraging activity between treatments, the minimum mass for each day of each colony was used to represent the peak of foragers leaving the colony. For foraging efficiency and nectar flow, we used the cumulative mass change per day. For each variable, an analysis was done with a LMM, with interaction between treatment and cold shock (pre and post) and colony as subject for repeated measurements. The foraging activity data based on the minimum mass were correlated (Pearson correlation) to foraging activity data from bee counters. Data were ln-transformed for both variables, and the absolute values were used for the mass. Zero values were removed for the analysis. We found a positive correlation between foraging activity estimated from mass and foraging activity estimated from bee counters (Fig. 1) (Pearson correlation: *r*=0.53, *n*=492, *P*<0.001), suggesting that the mass data were indeed a representation of foraging activity.

We explored the potential relationship between brood size and nurses by testing the correlation (Pearson correlation) between the number of brood cells and the number of nurses. The number of nurses was estimated by colony size×the fraction of nurses. See Supplementary Materials and Methods and Fig. S1 for colony size measurements. To estimate nurse effectivity, we used nurse load as a proxy, which was calculated by dividing the number of nurses by the number of brood cells. Nurse effectivity between *Varroa* and control colonies was compared by using a Mann–Whitney *U*-test.

All statistical analyses were done using R version 4.3.1.

## RESULTS

### Treatment validation

**Fig. 2.**
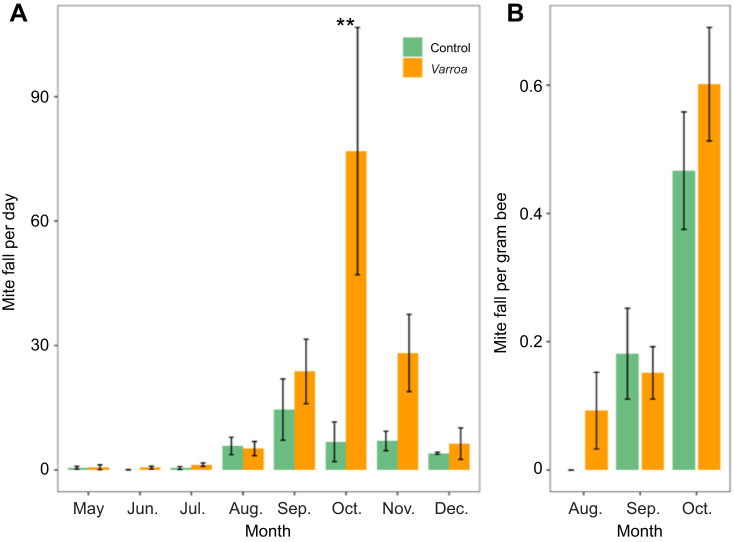
**Mean difference in mite infestation levels between control and *Varroa* colonies.** (A) Mean (±s.e.m.) daily mite fall over months in the different treatments (control *n*=5, *Varroa n*=5) for colonies in classical hives (CH). Mite infestation levels were estimated each month per colony by counting naturally falling mites. (B) Mean (±s.e.m.) number of mites per gram bee in the different treatments (control *n*=4, *Varroa n*=5) for colonies in observation hives (OH). Mite infestation levels were estimated each month per colony by sampling bees. Asterisks represent significant differences within months (**P<0.005). See Results for statistics.

In CH colonies, the mean daily mite fall in *Varroa* colonies was higher from September onwards compared with that of control colonies (daily mite fall was log+0.05 transformed, LMM: treatment×month *F*_7,60_=2.36, *P*=0.03). In mid-November, three colonies from the *Varroa* group died and were not included in the analysis for December, which may explain the marked decrease of *V. destructor* levels in *Varroa* colonies, as the most heavily infested colonies may have died first (Fig. 2A). Although there was no significant difference between OH colonies (number of mites per gram bee was log+0.05 transformed, LMM: treatment *F*_2,26_=1.60, *P*=0.2), the colonies in the *Varroa* group had higher levels of mite infestation in October (Fig. 2B), which is usually around the time when mite levels increase in colonies (Van Dooremalen et al., 2012). Typically, the method for mite counting used for OH colonies requires 300 bees (Dietemann et al., 2013), but we only collected 50 bees because of the smaller size of OH colonies. The small sample size for OH colonies may have led to an under-representation of the actual mite levels. It was not possible to use more bees per sample because of the small size of the colonies (three frames), especially considering the relatively high frequency of sampling. We did find a significant effect of month, where mite levels increased over time (*F*_2,26_=26.48, *P*<0.001).

### Demography of workers in the colony

**Fig. 3.**
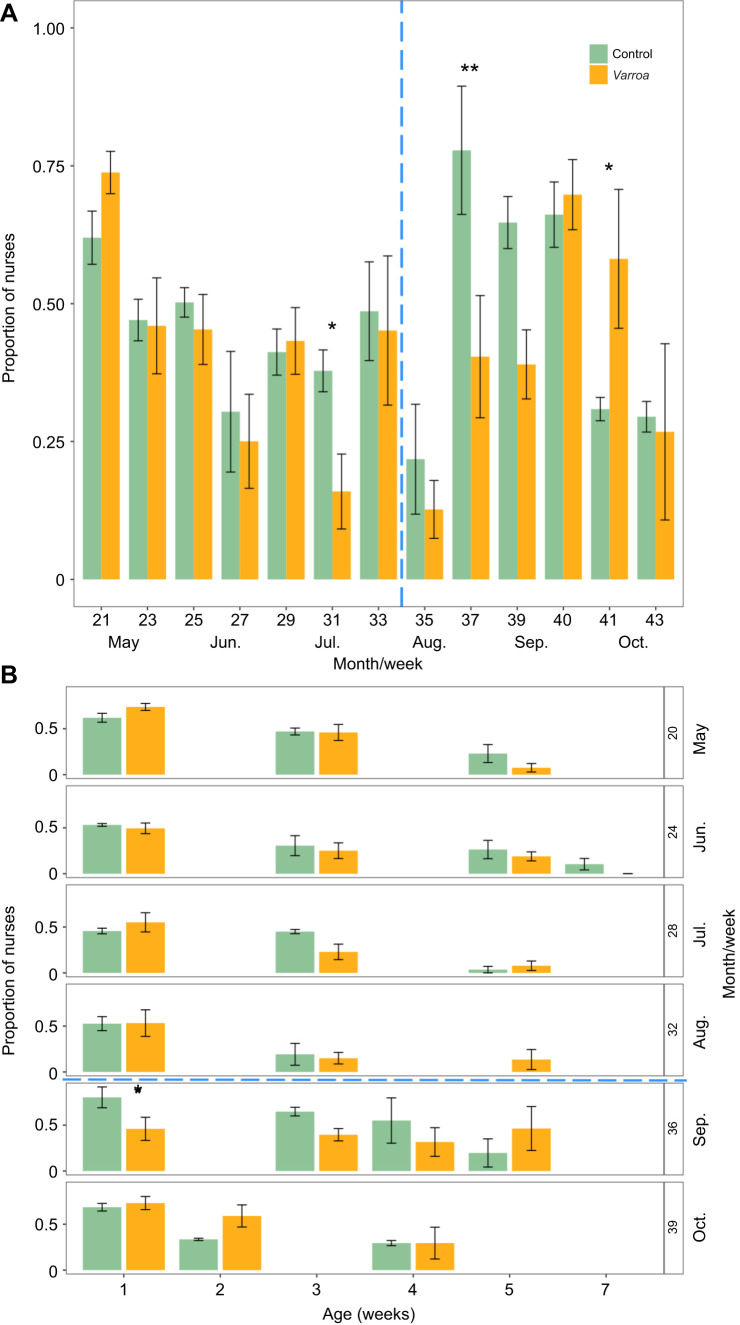
**Differences in the mean proportion of nurses in control and *Varroa* colonies over time and by age.** (A) Estimated mean (±s.e.m.) proportion of nurses over time, per treatment per week. The vertical dashed blue line represents cold shock (week 34). (B) Mean (±s.e.m.) proportion of nurses for each cohort, representing the week they were marked (week of emerging from brood) divided by the week they were counted (cohort age) per treatment. The horizontal blue dashed line represents cold shock (week 34). Control, *n*=5; *Varroa*, *n*=5. Asterisks indicate significant differences within weeks (*P<0.05, **P<0.005). See Results for statistics.

Chronic stress had an impact on the proportion of nurses in the colony (LMM: treatment×week *F*_12,115_=9.29, *P*=0.01), specifically after the cold shock experiment. For several weeks post-cold shock, control colonies had a markedly higher proportion of nurses compared with *Varroa* colonies (Fig. 3A), and there was a significant difference in week 37 (*P*=0.003). Conversely, *Varroa* colonies had a higher proportion of nurses after week 37 (Fig. 3A), showing a significant difference in week 41 (*P*=0.03). There was one week where the proportion of nurses differed between the two treatments pre-cold shock (week 31, *P*=0.04), and there were minor shifts in some weeks, but there was no clear pattern for these weeks. We also found an effect of marking week on the proportion of nurses (LMM: marking week×weeks alive×treatment *F*_9,156_=2.01, *P*=0.04). For cohorts that were marked after the cold shock (bees that were exposed to the cold shock while pupating), there was a notably higher proportion of nurses in control colonies compared with *Varroa* colonies (*P*=0.02) (Fig. 3B).

### Rate of task switching and survival

Overall, there was accelerated task switching in stressed colonies after cold shock. In CH colonies pre-cold shock, there was no significant difference in the rate of task switching from nurses to MABs between control and *Varroa* colonies (CPHM: *W*=2.129, *P*=0.07). However, we found that nurses in *Varroa* colonies post-cold shock were 24% more likely to switch earlier to MABs compared with those in control colonies. Pre-cold shock (until mid-August), the probability of switching tasks earlier increased over time by 1% (CPHM: *W*=15.556, *P*<0.001). This trend altered post-cold shock: until 13 days after (end of August), the probability of becoming a MAB increased over time by 5% (CPHM: *W*=10.844, *P*<0.001), then decreased by 5% (CPHM: *W*=−14.550, *P*<0.001) until 26 days after (mid-September), and finally increased by 11% (CPHM: *W*=8.491, *P*<0.001) until the end of the measurements (October).

In OH colonies, there was no difference between *Varroa* and control colonies in switching from nurses to MABs, both pre-cold shock (CPHM: *W*=−0.631, *P*=0.5) and post-cold shock (CPHM: *W*=0.9, *P*=0.9). Over time, the likelihood of switching from nurse to MAB increased by 3% (CPHM: *W*=7.411, *P*<0.001) pre-cold shock, and by 2% post-cold shock (CPHM: *W*=0.142, *P*=0.001). For the transition from MABs to foragers in OH colonies, however, cohorts from *Varroa* colonies were 40% more likely to switch earlier compared with cohorts from control colonies post-cold shock (CPHM: *W*=1.952, *P*=0.05). The probability of transitioning into a forager also initially increased over time, by 9% until cold shock (CPHM: *W*=6.858, *P*<0.001), and after cold shock by 5% until end of August (CPHM: *W*=4.173, *P*<0.001). From September onwards, time had no effect on the likelihood of becoming a forager (*W*=0.913, *P*=0.36).

Chronic stress had an effect on the survival of bees, which was more pronounced post-cold shock. Before cold shock, the cohorts from CH colonies from the *Varroa* treatment were 25% more likely to die compared with bees from control colonies (CPHM: *W*=10.15, *P*<0.001). After cold shock, the probability of dying for cohorts in *Varroa* colonies increased to 45% (CPHM: *W*=9.791, *P*<0.001). The likelihood of survival decreased over time, by 1% pre-cold shock (CPHM: *W*=31.09, *P*<0.001) and by 2% post-cold shock (CPHM: *W*=21.135, *P*<0.001). See Supplementary Materials and Methods and Fig. S3 for details on task switching and survival.

### Foraging activity and efficiency

**Fig. 4.**
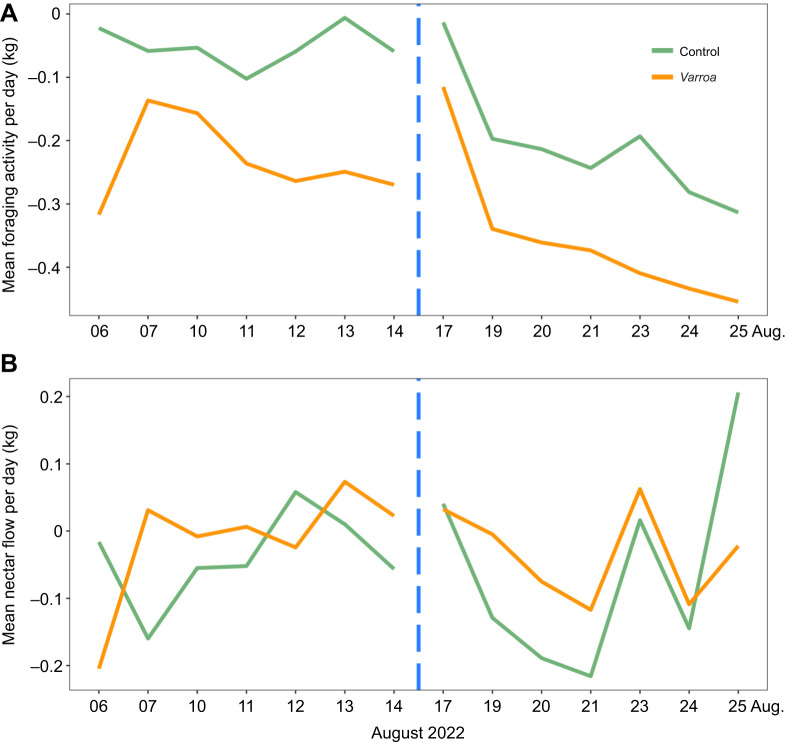
**Difference in foraging activity and efficiency in control and *Varroa* colonies pre- and post-cold shock.** (A) Mean foraging activity over different days per treatment. Foraging activity was calculated with mass data of each colony, by subtracting the raw mass at each time point from the mass at midnight, and then using the minimum value between dusk and dawn to represent the large-scale departure of foragers from the colony. Lower values indicate higher foraging activity. (B) Mean nectar flow, used as a proxy for foraging efficiency, per treatment for each day. Nectar flow was estimated by using the cumulative mass change per day. The vertical dashed blue line represents the timing of the cold shock. Control, *n*=5; *Varroa*, *n*=5. See Results for statistics.

Chronic stress and cold shock had an effect on the foraging activity of colonies. In CH colonies, *Varroa* colonies had higher foraging activity compared with control colonies, as seen by the greater decrease in daily mass (Fig. 4A), indicating that more foragers were leaving the colony (LMM: treatment *F*_1,6_=8.6, *P*=0.02, *Varroa* estimate=−0.292 g, s.e.=0.043 g; control estimate=−0.140 g, s.e.=0.048 g). Overall in all colonies, foraging activity was higher post-cold shock compared with that pre-cold shock (cold shock *F*_1,72_=11.7, *P*=0.001, pre-cold estimate=−0.143 g, s.e.=0.044 g; post-cold estimate=−0.289 g, s.e.=0.034 g). Weather data were used to confirm that the difference in foraging activity was indeed an effect of the cold shock and not due to differences in temperature, irradiation and rain over time (see Supplementary Materials and Methods and Fig. S2). However, treatment and cold shock had no influence on overall foraging efficiency as nectar flow was similar in the two time periods and also between *Varroa* and control colonies, as seen by the cumulative daily mass gain/loss (Fig. 4B) (LMM: treatment *F*_1,5_=0.16, *P*=0.7, cold shock *F*_1,78_=0.06, *P*=0.8).

### Number of nurses and number of brood cells

**Fig. 5.**
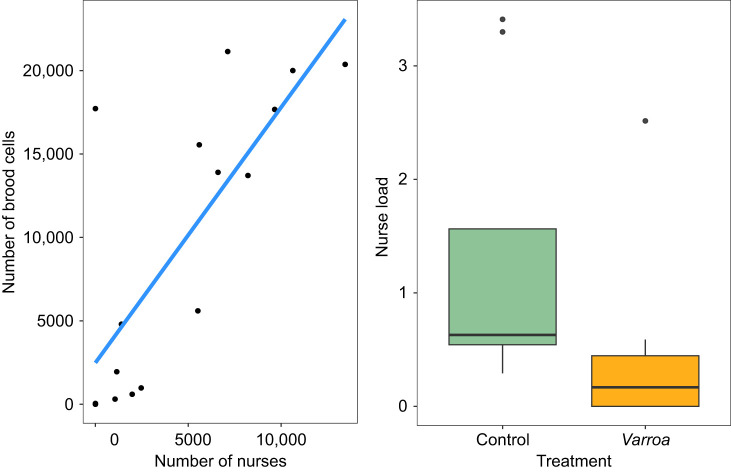
**Relationship between the number of brood cells and the number of nurses.** (A) Correlation between the estimated number of nurses and number of brood cells (Pearson correlation: *r*=0.77, *n*=16, *P*<0.001). The number of brood cells was estimated in July and October of 2022. (B) Difference in median nurse load, a proxy for nurse effectiveness, in control and *Varroa* colonies (Mann–Whitney *U*=68, *P*=0.01). Nurse load was estimated by the number of nurses per brood cell.

We found a positive relationship between the number of nurses and the number of brood cells (Pearson correlation: *r*=0.77, *n*=16, *P*<0.001) (Fig. 5A). There was also a significant difference between the nurse load of the control and *Varroa* colonies (Mann–Whitney *U*=68, *P*=0.01), where there were fewer bees per brood cell in *Varroa* colonies compared with control colonies (Fig. 5B), suggesting reduced nurse effectiveness.

## DISCUSSION

Here, we investigated how stress impacts DOL in honeybee colonies. Stressors such as *V. destructor* cause higher worker mortality (Van Dooremalen et al., 2012), earlier recruitment to the foraging task (Janmaat and Winston, 2000) and accelerated task switching (Zanni et al., 2018), possibly also resulting in a higher proportion of foragers compared with nurses (Ulgezen et al., 2021). We found that prior to the cold shock, worker survival in chronically stressed colonies was lower, but no major colony level changes were observed compared with control colonies. However, post-perturbation (cold shock), chronically stressed colonies had a lower proportion of nurses, higher worker mortality and earlier task switching. Regardless of an earlier shift to foraging, there was little change in overall foraging efficiency, as indicated by the similar nectar flow across treatments and periods. This is probably due to the increased foraging activity compensating for reduced individual efficiency. Yet, we conclude that chronic stress combined with cold shock appeared to exceed colonies' social resilience, shifting demography and impairing DOL.

It is known that honeybee colonies can adapt to changing conditions via DOL (Huang and Robinson, 1996), which is hypothesized to enhance their social resilience under stress (van Dooremalen et al., 2018). Previous research indicates that colonies manage acute stressors through DOL without any colony-level effects (Bordier et al., 2017; van Langevelde et al., 2020). Similarly, DOL in ants facilitates reorganization of workers after disturbances, promoting resilience (Sendova-Franks and Franks, 1994). However, we found that in chronically stressed colonies following a disturbance, DOL can shift, suggesting a loss of resilience. Pre-cold shock, demography and task-switching rates were similar between control and chronically stressed colonies. Despite the shorter lifespans in *V. destructor* colonies, we hypothesize that workers may have served as buffers against stress (Kolmes and Winston, 1988), without detrimental effects on task performance (Kolmes, 1985). If so, previously inactive workers become active after removal of active individuals (Charbonneau et al., 2017), enhancing colony flexibility. Yet, this has its limits as, once removed, inactive workers are not replenished (Charbonneau et al., 2017). The additional stress of cold shock (together with chronic *V. destructor* stress) is thought to exceed the social resilience capacity, impairing DOL and altering demographic patterns. While we cannot exclude the direct effects of *V. destructor*, the collapse of the majority of stressed colonies during winter suggests costs on a colony level, further supporting that there might be a loss of resilience.

The observed shift in DOL distribution is expected to result from sub-lethal and lethal effects of chronic stress on individual bees, during brood development and/or adulthood. Consistent with previous studies (Dainat et al., 2012; Van Dooremalen et al., 2012), we observed reduced worker survival in *V. destructor* colonies, which was exacerbated post-cold shock. We further show that task switching accelerated not only from MABs to foragers but also from nurses to MABs post-cold shock. In response to stress, bees usually begin foraging earlier (Dussaubat et al., 2013; Janmaat and Winston, 2000; Winston and Fergusson, 1985) and models suggest that this is driven by higher mortality and diminished efficiency in precocious foragers. In line with this, we found that foragers and nurses in *V. destructor* colonies were less efficient. Despite increased foraging activity in *V. destructor* colonies, particularly post-cold shock, no differences were observed in daily mass gain/loss, our proxy for pollen and nectar inflow, between treatments. Similarly, nurses in *V. destructor* colonies had higher workloads, potentially reducing their effectiveness, as low worker-to-larvae ratio is linked to higher offspring mortality (Eischen et al., 1983). Nurse inefficiency has long-lasting effects, such as impairing the formation of long-lived winter bees, which are crucial for winter survival. Typically, long-lived winter bees are produced around the time of our cold shock experiment (mid-August) (Van Dooremalen et al., 2012). However, post-cold shock, the timing of worker emergence negatively impacted bee survival.

We found that the proportion of nurses was especially low for the initial set of workers that emerged post-cold shock (pupating during cold shock), indicating that brood development and mortality were also affected. Perturbations in stressed colonies diminish thermoregulation ability (Ulgezen et al., 2024), which is essential for healthy brood development. Cold stress during pupal development is found to negatively impact adult fitness and survival (Wang et al., 2016), and harsh conditions can lead to brood cannibalization (Schmickl and Crailsheim, 2001). The positive link that we found between the number of brood cells and the number of nurses suggests that the reduction in nurses is caused by a decrease in brood size.

Interestingly, while chronically stressed colonies initially shifted toward a higher proportion of foragers post-cold shock, this trend reversed a few weeks later, where the proportion of nurses drastically increased. Brood rearing activities in colonies have been found to increase as a result of stress-induced mortality (Schott et al., 2021), thereby possibly increasing the number of nurses. This subsequent reversal in nurse proportion is hypothesized to be a compensatory mechanism for the initial loss of workers and/or brood.

Supporting previous research (Ulgezen et al., 2021; van Dooremalen et al., 2018), our results indicate that honeybee colonies initially adapt to stress by maintaining flexibility in task allocation. Honeybee colonies are highly plastic to worker mortality in times of high resource availability, i.e. spring and summer (Lemanski et al., 2020), and therefore more resilient to stress. Loss of resilience is thought to become more apparent and have more immediate fitness implications under harsh conditions with limited resources (Ulgezen et al., 2024). However, we found that there was a higher loss of workers and accelerated task switching after the cold shock in chronically stressed colonies, shifting the demography of the colony towards a higher proportion of foragers. Therefore, we conclude that chronic stress exposure leaves colonies more vulnerable to disturbances, ultimately compromising social resilience due to suboptimal worker composition and condition. Our results contribute to a deeper understanding of DOL and stress response in social insect colonies.

## Supplementary Material

10.1242/jexbio.247976_sup1 Supplementary information
