# Supplementary material for: Shift in distribution of division of labour in chronically stressed honeybee colonies after perturbation
Source: J Exp Biol. 2024 Nov 8;227(21):jeb247976. doi: 10.1242/jeb.247976 (PMC11574360; doi:10.1242/jeb.247976)
Supplement: Supplementary information [file jexbio-227-247976-s1.pdf]

Supplementary Materials and Methods

In this supplementary material we included additional methods and results, supporting the main article. There is information on:

- Colony size measurements for the main experiment (colonies in classical hives)
- Differences in weather conditions before and after cold shock and its potential influence on foraging activity
- Figures for the results of task switch and survival analysis with Cox Proportional Hazards Model

Colony size

Every two to four weeks, depending on weather conditions, colony size was estimated in classical hive (CH) colonies. This was done by taking a photo of the top side of the hive, following the same methods described in van Dooremalen et al. (2018). Colony size was calculated based on the fraction of the area of bees, namely by dividing the area occupied with bees by the total area available. For reference, a fraction of one represents approximately 18.000 bees (van Dooremalen et al., 2018). Frequency of measurements was increased during the cold shock experiment (week before, week of and week after) to better capture the possible effects. For the analysis, we used LMM with interaction term treatment x week and included colony as subject for repeated measurements. As we did not find a significant interaction, this term was removed. While there was no significant effect of treatment (LMM: $F_{1,10}$ =1.76, p = 0.214), the colony size was slightly higher in control colonies for most of the year compared to *V. destructor* colonies (Figure S1). We did find a significant effect of week where beginning of the bee season foraging season, i.e. spring, (LMM: $F_{1,12}$ =49.6, p < 0.001), the colony size drastically increased, and then decreased in winter (Figure S3). There were no significant differences between the weeks around cold-shock, so we presume that it did not have an effect on colony size.

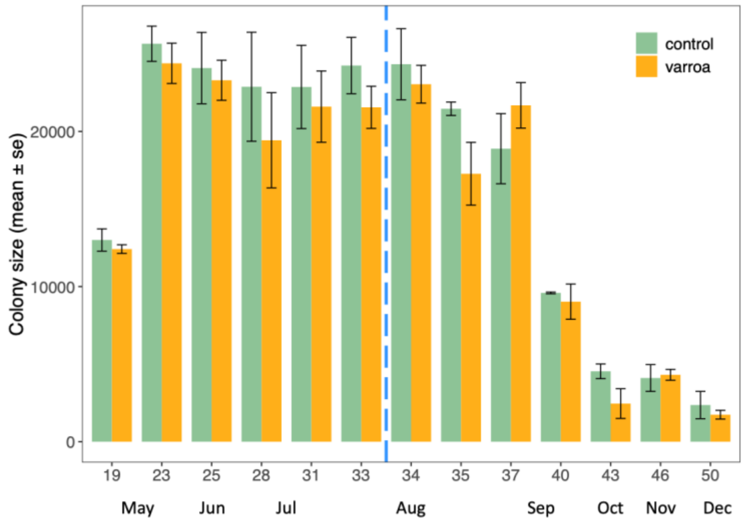

Fig. S1. Mean difference in colony size between presumably non-stressed (control) and high *V. destructor* infested (varroa) colonies over weeks. Vertical line represents time of cold shock.

Journal of Experimental Biology • Supplementary information

Weather effects on foraging activity

A local weather station (Weatherhelix, Barani design) that recorded measurements in 10-min intervals, was placed at the apiary. We used ambient temperature (°C) and irradiation (W/m^2), to see whether there was a difference in weather the week before and after cold shock, concerning the days that were used in the analysis of foraging activity. Temperature and solar radiation have been found to be the most influential local weather variables on foraging activity (Clarke and Robert, 2018). As there was no rainfall during the measurement days, we did not use it for the analysis. The difference in weather pre- and post-cold shock was tested with a paired t-test, using mean irradiation and mean temperature per day. The mean temperature did not differ between pre (21.6±1.4 °C) and post (20.5±1 °C) cold shock (paired t-test: t_6 = -0.82, p = 0.4) (Figure S2a). Interestingly, there was a significant difference in mean irradiation, where it was higher pre-cold shock (159±4 W/m^2) compared to post-cold shock (98±10 W/m^2; paired t-test: t_6 = -5.56, p = 0.001) (Figure S2b). As we found that foraging activity increased post-cold shock, and as there is a positive influence of solar radiation levels on foraging level (Clarke and Robert, 2018), our results indicate that this increase in foraging activity is not a result of a change in weather conditions.

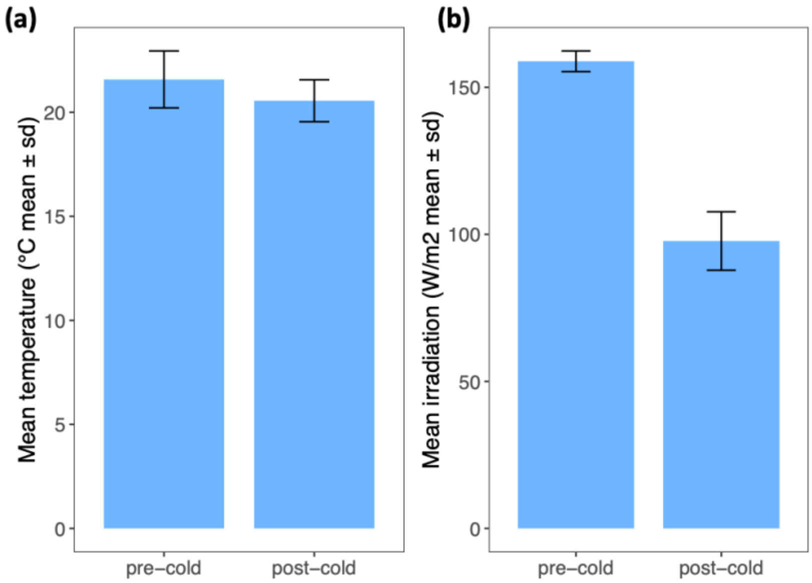

Fig. S2. Average weather conditions 7 days prior and 7 days after the cold shock. (a) Mean difference in daily average temperature pre- and post-cold shock (b) Mean difference in daily average irradiation pre- and post- cold shock.

Task switch and survival

Analysis on the difference between rate of task switching and survival time of workers from control and chronically stressed, high *V. destructor* infested, colonies was done with Cox Proportional Hazards Model. More information on methods and statistical results are available in the main article. Figures of results are represented here (Figure S3).

Journal of Experimental Biology • Supplementary information

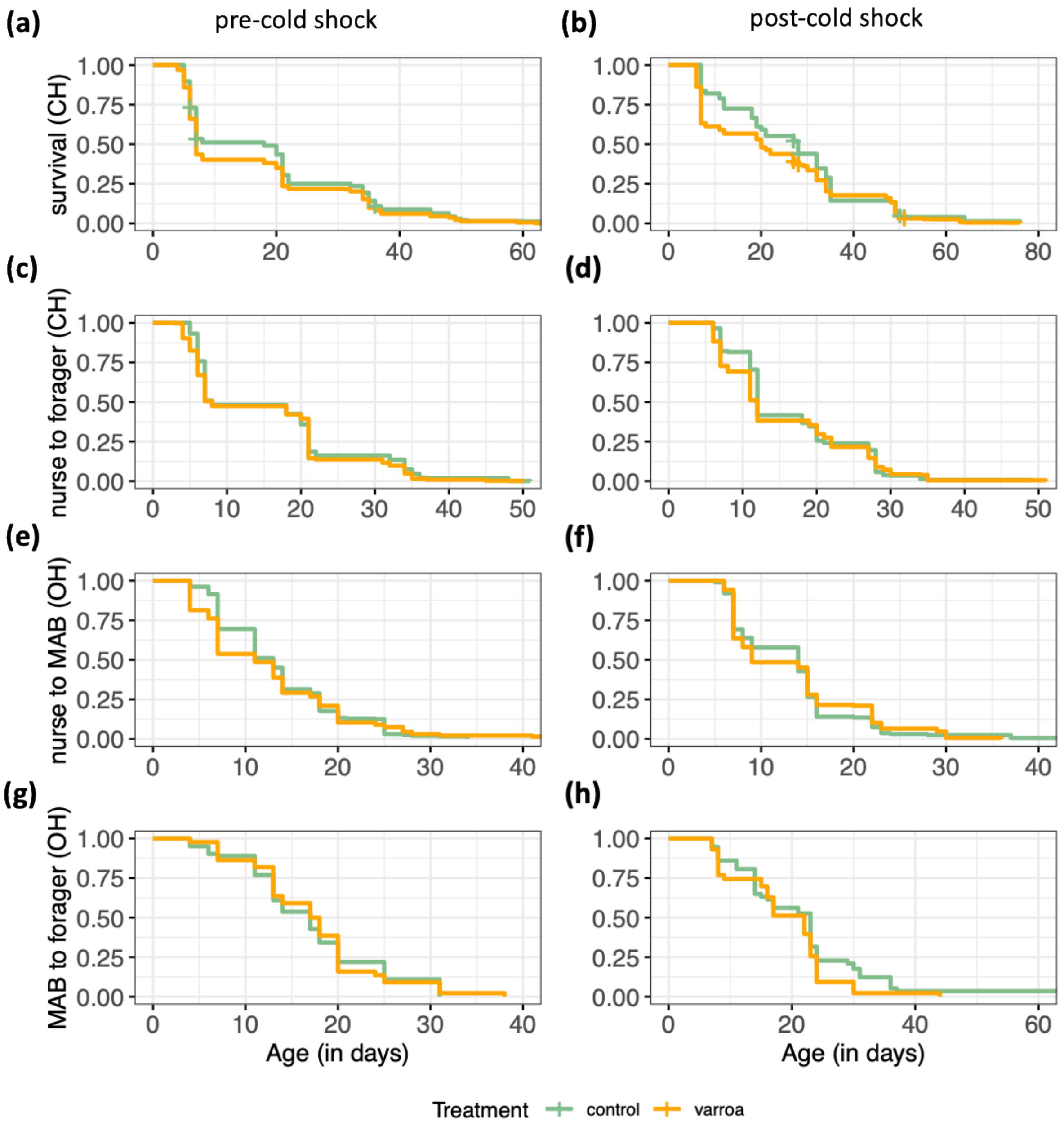

Fig. S3. Cox proportional hazard curves for presumably non-stressed (control) and high *V. destructor* infested (varroa) colonies. Figures only show treatment effect. (a-b) Difference in survival between cohorts from control and varroa colonies in classical hives (CH) pre- and post- cold shock. (c-d) Difference in rate of task switching from nurses to foragers in control and varroa colonies in CHs pre- and post-cold shock. (e-f) Difference in rate of task switching from nurses to MABs in control and varroa colonies in observation hives (OH) pre- and post-cold shock. (g-h) Difference in rate of task switching from MABs to foragers in control and varroa colonies in OHs pre- and post- cold shock. See main article for methods and statistics.

Journal of Experimental Biology • Supplementary information

References

Clarke, D. and Robert, D. (2018). Predictive modelling of honey bee foraging activity using local weather conditions. *Apidologie* **49**, 386-396.

van Dooremalen, C., Cornelissen, B., Poleij-Hok-Ahin, C. and Blacquière, T. (2018). Single and interactive effects of Varroa destructor, Nosema spp., and imidacloprid on honey bee colonies (Apis mellifera). *Ecosphere* **9**, e02378.

Journal of Experimental Biology • Supplementary information
